# Supplementary material for: Aspirin Inhibition of Prostaglandin Synthesis Impairs Mosquito Egg Development
Source: Cells. 2022 Dec 16;11(24):4092. doi: 10.3390/cells11244092 (PMC9776805; doi:10.3390/cells11244092)
Supplement: Supplementary file 1 [file cells-11-04092-s001.zip › cells-2062172-supplementary.pdf]

Supplementary Materials

Table S1. Primers used in this study

Table S2. GenBank accession numbers of different PLA_2 genes to construct phylogenetic tree of Figure S2A

Figure S1. Effects of different concentrations of aspirin ('ASP') on choriogenesis of *Ae. albopictus*. (**A**) Hemocoelic injection. ASP was injected into 5 days old females 10 min before blood feeding ('BF'). At 72 h post BF, ovaries were collected from treated females in PBS to count the number of chorionated oocytes. (**B**) Oral feeding. Different concentrations of ASP were prepared with a 10% sugar solution. The sugar-ASP solution (5 mL) was provided to an adult cage containing 10 females at 3 h before BF. After BF, females were allowed to continuously feed the sugar-ASP solution for 3 days and then assessed for the development of chorionated oocytes. Each treatment used 10 females. Different letters above standard error bars indicate significant differences among means at Type I error = 0.05 (LSD test).

Figure S2. Identification of six *PLA_2* genes of *Ae. albopictus.* (**A**) Phylogenetic tree analysis of *Aa-PLA_2*s. The tree was generated by the Maximum likelihood tree method using software package MEGA6.0, where evolutionary distances were computed using the Poisson correction method. Amino acid sequences of different PLA_2s were retrieved from GenBank with accession numbers of Table S2. (**B**) Typical functional domains of six *Aa-PLA_2*s. Domains were predicted using Prosite (https://prosite.expasy.org/) and SMART protein (http://smart.embl-heidelberg.de/).

Figure S3. Prediction of peroxidases (POXs) associated with PG biosynthesis in *Ae. albopictus*. (**A**) Phylogenetic tree analysis of mosquito POXs. The tree was generated by the Maximum likelihood tree method using software package MEGA-X, where evolutionary distances were computed using the Poisson correction method. Amino acid sequences of different peroxidases were retrieved from GenBank with accession numbers indicated along with individual gene names in the tree. (**B**) Domain analysis of five Aa-POXs clustered with known COX-POXs.

Figure S4. Phylogenetic tree and domain analyses of *Aa-Shade*. (**A**) Phylogenetic analysis of *Shade* genes from different insects. The analysis was performed using MEGA-X. Bootstrapping values were obtained with 1,000 repetitions to support branching and clustering. Amino acid sequences of selected genes were retrieved from GenBank with accession numbers indicated along with individual gene names in the tree. (**B**) Domain analysis of Aa-Shade. 'SP' and 'HBD' stand for the signal peptide and heme-binding

domain, respectively. Domains were predicted using Prosite (https://prosite.expasy.org/) and SMART protein (http://smart.embl-heidelberg.de/).

Figure S5. RNAi of three *PLA2* (*Aa-PLA2-IIA*, *Aa-PLA2-IIIB*, *Aa-PLA2-VI*) and three *POX* (*Aa-POX19*, *Aa-POX20*, *Aa-POX15*) genes. RNAi was performed by injecting 100 ng of gene-specific dsRNA or control dsRNA (‘dsCON’) to 5 days old females at 24 h before blood-feeding (BF). Changes in mRNA levels were assessed at 72 h after injecting dsRNA or dsCON by RT-qPCR using *RpS6* as a reference gene to normalize target gene expression level. Each treatment was replicated three times with independent tissue preparations. Asterisk (*) above standard error bars indicates significant difference among means at Type I error = 0.05 (LSD test).

Figure S6. Molecular characteristics of *Ae. albopictus* PGE_2 receptor (*Aa-PGE2R*: GenBank accession number: XP_029736412.1). (**A**) Domain analysis of *Aa-PGE2R*. A cleavage site of signal peptide (23 residues) is denoted by scissor. Seven transmembrane domains are marked on phospholipid bilayer with locations. N-glycosylation sites are denoted by an orange circle and phosphorylation sites are marked by a blue triangle. Domains were predicted using Prosite (https://prosite.expasy.org/) and SMART protein (http://smart.embl-heidelberg.de/). (**B**) A phylogenetic analysis. The tree was generated by the Maximum likelihood tree method using software package MEGA6.0, where evolutionary distances were computed using the Poisson correction method. Amino acid sequences of different PGE_2R were retrieved from GenBank with accession numbers of KAB1277535.1 for *C. dromedarius* (DP2), NP_000944.1 for *H. sapiens* (DP2), NP_071577.1 for *R. norvegicus* (DP2), XP_021258611.1 for *N. meleagris* (DP2), AAI12966.1 for *H. sapiens* (FP), JAA36212.1 for *P. troglodytes* (FP), AAZ08074.1 for *B. bubalis* (FP), EDL82480.1 for *R. norvegicus* (FP), NP_032993.2 for *M. musculus* (IP), NP_001015622.2 for *B. Taurus* (IP), EDM08294.1 for *R. norvegicus* (IP), XP_005361096.1 for *M. ochrogaster* (IP), NP_001345441.1 for *M. musculus* (TXA), NP_001161391.1 for *B. Taurus* (TXA), JAA33130.1 for *P. troglodytes* (TXA), AAH74749.1 for *H. sapiens* (TXA), NP_000946.2 for *H. sapiens* (EP1), NP_001265404.1 for *R. norvegicus* (EP1), NP_038669.1 for *M. musculus* (EP1), NP_001179077.1 for *B. taurus* (EP1), NP_000947.2 for H. sapiens (EP2), NP_112350.1 for *R. norvegicus* (EP2), NP_032990.1 for *M. musculus* (EP2), NP_777013.1 for *B. taurus* (EP2), NP_942007.1 for *H. sapiens* (EP3), NP_036836.1 for *R. norvegicus* (EP3), NP_001346674.1 for *M. musculus* (EP3), NP_851375.1 for *B. taurus* (EP3), NP_000949.1 for *H. sapiens* (EP4), NP_114465.3 for *R. norvegicus* (EP4), NP_001129551.1 for *M. musculus* (EP4), and NP_777014.1 for *B. Taurus* (EP4).

Figure S7. Expression profile of *PGE2R* in *Ae. albopictus*. (**A**) Expression pattern in different developmental stages from egg to male and female adults. (**B**) Expression in different body parts of *Ae. albopictus* females at 3 days after blood-feeding (‘BF’): head (‘HD’), thorax (‘TH’), ovary (‘OV’), and abdomen (‘ABD’). (**C**) Relationship between the expression level of *Aa-PGE2R* and the number of chorionated oocytes after BF. Expression of actin, a reference gene, was used to normalize gene expression levels in RT-qPCR. All measurements were independently replicated three times.

Figure S8. Molecular characterization of *Ae. albopictus* vitellogenin protein (*Aa-Vg1* and *Aa-Vg2*). (**A**) A phylogenetic tree analysis. The tree was generated by the Maximum likelihood tree method using software package MEGA6.0, where evolutionary distances were computed using the Poisson correction method. Amino acid sequences of different vitellogenin genes were retrieved from GenBank. (**B**) Domain analysis of *Aa-Vg1* and *Aa-Vg2*. Domains were predicted using Prosite (https://prosite.expasy.org/) and SMART protein (http://smart.embl-heidelberg.de/). (**C**) Expression pattern of *Aa-Vg1* and *Aa-Vg2* after blood-feeding (BF).

Table S1. Primers used in this study

Genes	Primer sequence (5' - 3')
Aa-PLA$_2$-IIIA	CAAGTGTGACCGAGATTTTC
	GTCTCAAAAACAGCCTGTAAG
Aa-PLA$_2$-IIIB	CCTGAAAATGTCCGACTCC
	TTTCCTGTTGTCACGGATG
Aa-PLA$_2$-VI	TCAAAACTGTACGGGAAGAG
	CACATCGATCGAGTTTTTCC
Aa-PLA$_2$-VIII	TTCACCGGACAAAGTGATAG
	ATCGAACAGCTCGAAAATCT
Aa-PLA$_2$-XII	ACATCCCACAATCGGATGGG
	TGGACATTTGAATATGCAAT
	TAATACGACTCACTATAGGGAGAACATCCCACAATCGGATGGG
	TAATACGACTCACTATAGGGAGATGGACATTTGAATATGCAAT
Aa-PLA$_2$-XV	CCGATATCAACAAACCGAAC
	TTCCGTTGTAGTTACCCTTG
Aa-Shade	AACAATATGACCCACCATCC
	GCTTGTAGACGTTTTGGTTT
	TAATACGACTCACTATAGGGAGAAACAATATGACCCACCATCC
	TAATACGACTCACTATAGGGAGAGCTTGTAGACGTTTTGGTTT
Aa-POX4	GTCCTTACAACGATTACCGA
	AAAATAGTACCGATCGCCTC
Aa-POX9	ATGCCGAAATCAAACACTTC
	TACAATGACTGTAGCTTGGC
	TAATACGACTCACTATAGGGAGAATGCCGAAATCAAACACTTC
	TAATACGACTCACTATAGGGAGATACAATGACTGTAGCTTGGC
Aa-POX15	CTTAATACTCCTCGACCGAC
	TCAAAGAGTGCTCAAAGTCT
	TAATACGACTCACTATAGGGAGACTTAATACTCCTCGACCGAC
	TAATACGACTCACTATAGGGAGATCAAAGAGTGCTCAAAGTCT
Aa-POX19	GACCTTACAACGACTACCTC
	TGCTGTAAAAGTACCGATCC
	TAATACGACTCACTATAGGGAGAGACCTTACAACGACTACCTC
	TAATACGACTCACTATAGGGAGATGCTGTAAAAGTACCGATCC
Aa-POX20	GTTTCTTCAGATTGCTCACC
	GGCTAGATCTGATCCGAAAG
	TAATACGACTCACTATAGGGAGAGTTTCTTCAGATTGCTCACC
	TAATACGACTCACTATAGGGAGAGGCTAGATCTGATCCGAAAG
Aa-Vg1	CCGTAGTCAAAATTCGAAGC
	ACTCTTCAGCGATATTCCAC
Aa-Vg2	CAAGCCACAGTATCCTTCTT
	CTCGTACTCATCAAATCCGT
Aa-PGE$_2$R	CCAACATGCACCACTTCT
	GGAACTGCCTTCGCATCT
	TAATACGACTCACTATAGGGAGACCAACATGCACCACTTCT
	TAATACGACTCACTATAGGGAGAGGAACTGCCTTCGCATCT
RpS6	ACAAGCTGCGTCACTTCTACGACA
	CTTGTCGTTTCCACCGGCAATCTT
	CACGTTCAGTCAGGATCTTC

Table S2. GenBank accession numbers of different PLA_2 genes to construct phylogenetic tree of Fig. S2A

Gene	Accession	Scientific name
Ame-III	XP_002915601.2	*Ailuropoda melanoleuca*
Ami-III	XP_006032459.1	*Alligator sinensis*
Av-X	XP_009862694.1	*Apaloderma vittatum*
Bt-XII	NP_001091532.1	*Bos taurus*
Cf-V	XP_006188390.1	*Camelus ferus*
Bt-III	NP_001074379.2	*Bos taurus*
Cf-X	XP_006179161.1	*Camelus ferus*
Cl-I	NP_001003320.1	*Canis lupus familiaris*
Cl-III	XP_025330373.1	*Canis lupus dingo*
Cp-I	XP_003477984.1	*Cavia porcellus*
Cp-X	XP_010001508.1	*Chaetura pelagica*
Cg-III	EGW07116.1	*Cricetulus griseus*
Dr-III	NP_001032489.1	*Danio rerio*
Eqc-I	XP_001489303.1	*Equus caballus*
Eh-X	XP_010154523.1	*Eurypyga helias*
Fc-III	XP_023097342.1	*Felis catus*
Fg-II	XP_009572314.1	*Fulmarus glacialis*
Gg-I	NP_001138961.1	*Gallus gallus*
Gg-X	ADG46026.1	*Gallus gallus*
Gs-X	XP_009813736.1	*Gavia stellata*
Hs-II	NP_000291.1	*Homo sapiens*
Hs-IIF	NP_073730.3	*Homo sapiens*
Hs-X	NP_003552.1	*Homo sapiens*
Hs-V	5245948.1	*Homo sapiens*
Hs-XII	NP_110448.2	*Homo sapiens*
Hs-III	NP_056530.2	*Homo sapiens*
It-III	XP_005339912.1	*Ictidomys tridecemlineatus*
Lc-XIIA	XP_010754065.1	*Larimichthys crocea*
Ls-XIIB	XP_021399547.1	*Lonchura striata domestica*
Mm-XIIA	NP_001181806.1	*Macaca mulatta*
Mn-III	XP_011748880.1	*Macaca nemestrina*
Lc-X	XP_017689444.1	*Lepidothrix coronata*
Ma-III	XP_005068966.1	*Mesocricetus auratus*
Md-III	XP_008543925.1	*Microplitis demolitor*
Mm-X	NP_036117.1	*Macaca mulatta*
Mm-IB	NP_035237.1	*Macaca mulatta*
Mm-II	NP_032894.2	*Macaca mulatta*
Mm-III	NP_766379.2	*Macaca mulatta*
Mm-V	P97391.2	*Macaca mulatta*
Mm-XII	NP_075685.2	*KAF1442899.1*
Nn-X	XP_009464949.1	*Nipponia nippon*
Oc-IB	Q7M334.1	*Oryctolagus cuniculus*
Pp-III	XP_003826977.1	*Pan paniscus*
Oa-IB	XP_004017463.1	*Ovis aries*
Pp-V	XP_034812955.1	*Pan paniscus*
Pl-X	KFQ64262.1	*Phaethon lepturus*

Dp-X	XP_009897154.1	*Dryobates pubescens*
Pa-III	XP_009215381.1	*Papio anubis*
Pg-X	XP_010076871.1	*Pterocles gutturalis*
Pa-X	XP_009323089.1	*Pygoscelis adeliae*
Rn-X	NP_058872.1	*Rattus norvegicus*
Rn-IB	NP_113773.1	*Rattus norvegicus*
Rn-IIC	NP_062075.1	*Rattus norvegicus*
Rn-IID	NP_001013446.1	*Rattus norvegicus*
Rn-III	NP_001099485.1	*Rattus norvegicus*
Rn-V	NP_058870.1	*Rattus norvegicus*
Rn-XIIA	EDL82189.1	*Rattus norvegicus*
Se-III	AZL90156.1	*Spodoptera exigua*
Te-X	KFV16621.1	*Tauraco erythrolophus*
Xl-XIIA	NP_001085480.	*Xenopus laevis*
Aa-XII	XP_029724479.1	*Aedes albopictus*
Dh-XII	XP_023164765.1	*Drosophila hydei*
Hsa-I	3ELO_A	*Homo sapiens*
Pt-I	XP_001160305.1	*Pan troglodytes*
Mm-I	NP_001343515.1	*Mus musculus*
Rn-I	NP_113773.1	*Rattus norvegicus*
Dre-I	NP_001107095.1	*Danio rerio*
Hsa-IV	NP_077734.2	*Homo sapiens*
Pt-IV	XP_001165694.1	*Pan troglodytes*
Ggg-IV	XP_004028114.1	*Gorilla gorilla gorilla*
Rn-IV	NP_598235.2	*Rattus norvegicus*
Mm-IV	NP_032895.1	*Mus musculus*
Hsa-VI	NP_001004426.1	*Homo sapiens*
Ggg-VI	XP_030861468.1	*Gorilla gorilla gorilla*
Poa-VI	XP_024095668.1	*Pongo abelii*
Mmu-VI	XP_028683552.1	*Macaca mulatta*
Pit-VI	XP_023077806.1	*Piliocolobus tephrosceles*
Hsa-VII	AAB04170.1	*Homo sapiens*
Pp-VII	XP_034818104.1	*Pan paniscus*
Ggg-VII	XP_004044188.1	*Gorilla gorilla gorilla*
Pt-VII	XP_009449675.1	*Pan troglodytes*
Aa-IIIA	XP_019933483.1	*Aedes albopictus*
Aa-IIIB	XP_029713527.1	*Aedes albopictus*
Aa-VIII	XP_029714924.1	*Aedes albopictus*
Aa-XV	XP_029720738.1	*Aedes albopictus*
Aa-VI	XP_029732314.1	*Aedes albopictus*
Rn-XV	NP_001004277.1	*Rattus norvegicus*
Hsa-VIII	NP_001242939.1	*Homo sapiens*
Pp-VIII	XP_003811265.1	*Pan paniscus*
Ggg-VIII	XP_030868609.1	*Gorilla gorilla gorilla*
Pat-VIII	XP_009452256.1	*Pan troglodytes*
Min-VIII	XP_016054620.1	*Miniopterus natalensis*
Mm-XV	NP_598553.1	*Mus musculus*
Hsa-XV	4X90_A	*Homo sapiens*
Op-XV	XP_004584230.1	*Ochotona princeps*

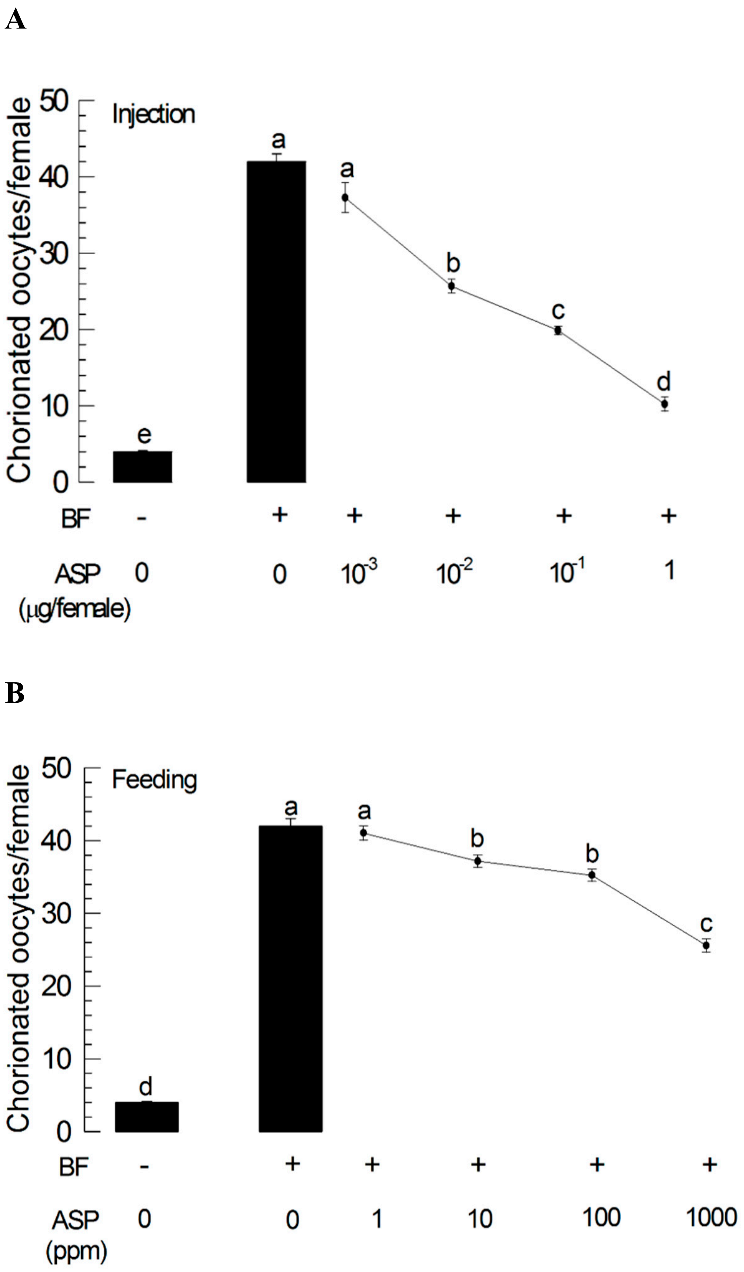
A
Injection
Chorionated oocytes/female
50
40
30
20
10
0
a
a
b
c
d
e
BF - + + + + +
ASP 0 0 10^{-3} 10^{-2} 10^{-1} 1
(µg/female)
B
Feeding
Chorionated oocytes/female
50
40
30
20
10
0
a
a
b
b
c
d
BF - + + + + +
ASP 0 0 1 10 100 1000
(ppm)

Figure S1

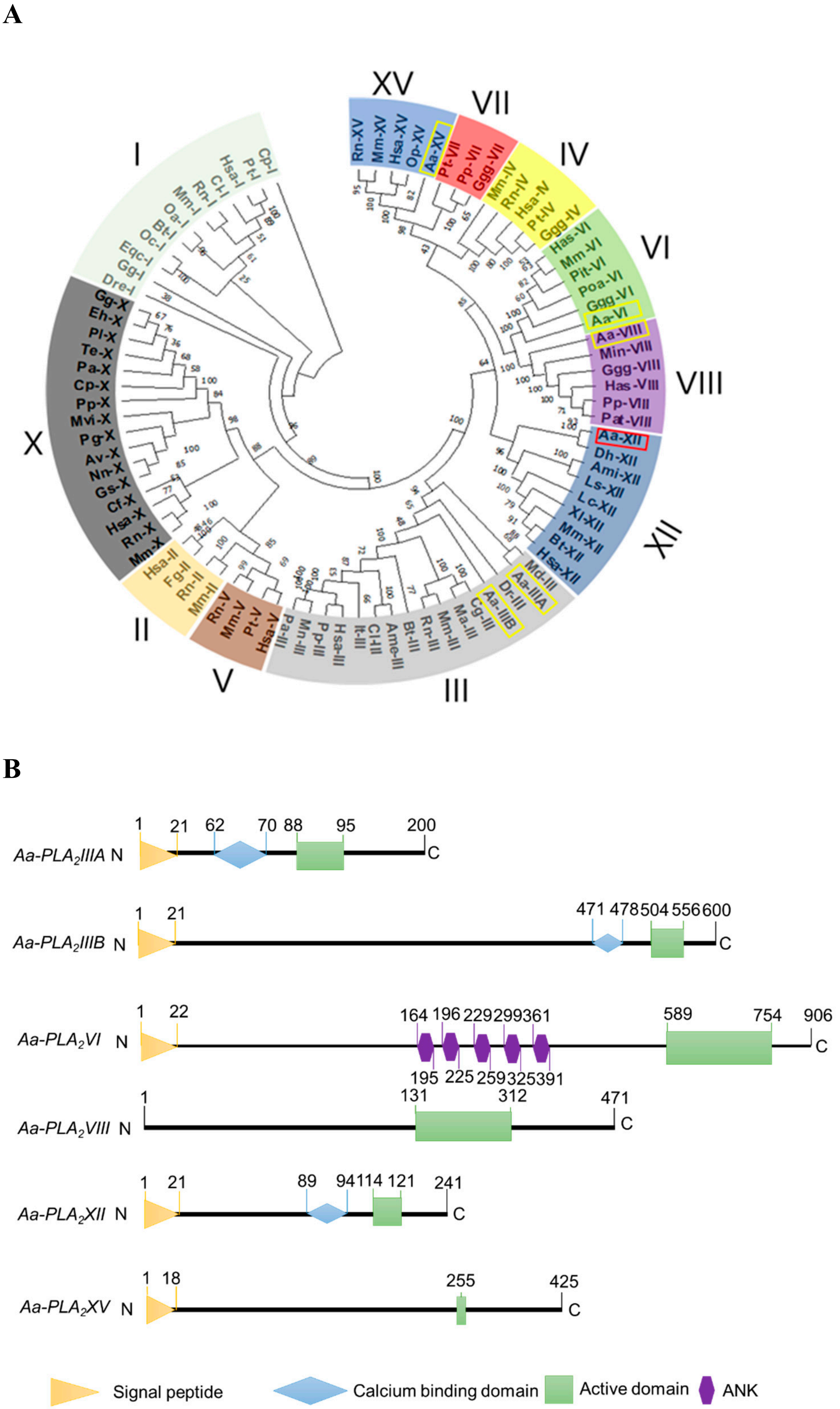
A
XV
VII
IV
VI
VIII
XII
III
V
II
X
I
Rn-XV
Mm-XV
Hsa-XV
Op-XV
Aa-XV
Pt-VII
Pp-VII
Ggg-VII
Mm-IV
Rn-IV
Hsa-N
Pt-IV
Ggg-IV
Has-VI
Mm-VI
Pit-VI
Poa-VI
Ggg-VI
Aa-VI
Aa-VIII
Min-VIII
Ggg-VIII
Has-VIII
Pp-VIII
Pat-VIII
Aa-XII
Dh-XII
Ami-XII
Ls-XII
Lc-XII
Xl-XII
Mm-XII
Bt-XII
Hsa-XII
Md-III
Aa-IIIA
Dr-III
Aa-IIIB
Cg-III
Ma-III
Mm-III
Rn-III
Bt-III
Ame-III
Cl-III
It-III
Hsa-III
Pp-III
Mn-III
Pa-III
Hsa-V
Pt-V
Mm-V
Rn-V
Mm-II
Rn-II
Fg-II
Hsa-II
Mm-X
Rn-X
Hsa-X
Cf-X
Gs-X
Nn-X
Av-X
Pg-X
Mvi-X
Pp-X
Cp-X
Pa-X
Te-X
Pl-X
Eh-X
Gg-X
Dre-I
Gg-I
Eqc-I
Oc-I
Bt-I
Oa-I
Mm-I
Rn-I
Cl-I
Hsa-I
Pt-I
Cp-I
B
Aa-PLA2IIIA N
1 21 62 70 88 95 200
C
Aa-PLA2IIIB N
1 21 471 478 504 556 600
C
Aa-PLA2VI N
1 22 164 196 229 299 361 589 754 906
195 225 259 325 391
C
Aa-PLA2VIII N
1 131 312 471
C
Aa-PLA2XII N
1 21 89 94 114 121 241
C
Aa-PLA2XV N
1 18 255 425
C
Signal peptide
Calcium binding domain
Active domain
ANK

Figure S2

A

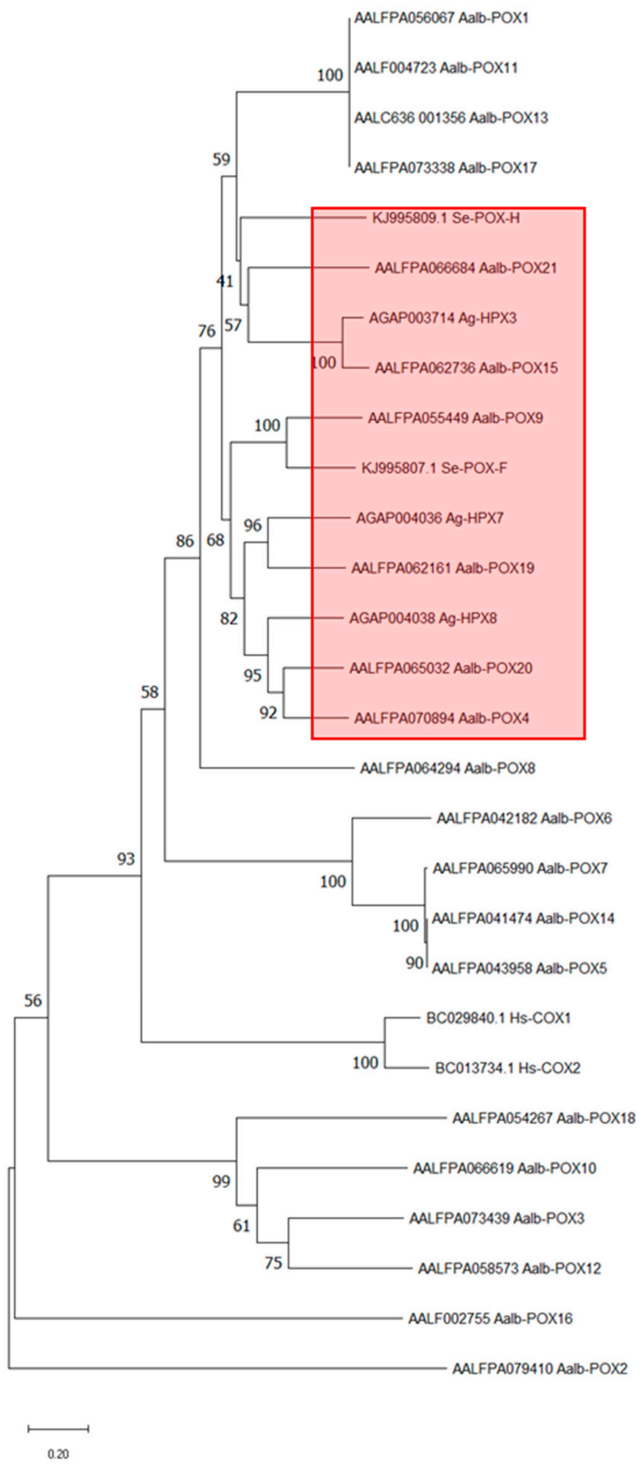
AALFPA056067 Aalb-POX1
AALF004723 Aalb-POX11
AALC636 001356 Aalb-POX13
AALFPA073338 Aalb-POX17
KJ995809.1 Se-POX-H
AALFPA066684 Aalb-POX21
AGAP003714 Ag-HPX3
AALFPA062736 Aalb-POX15
AALFPA055449 Aalb-POX9
KJ995807.1 Se-POX-F
AGAP004036 Ag-HPX7
AALFPA062161 Aalb-POX19
AGAP004038 Ag-HPX8
AALFPA065032 Aalb-POX20
AALFPA070894 Aalb-POX4
AALFPA064294 Aalb-POX8
AALFPA042182 Aalb-POX6
AALFPA065990 Aalb-POX7
AALFPA041474 Aalb-POX14
AALFPA043958 Aalb-POX5
BC029840.1 Hs-COX1
BC013734.1 Hs-COX2
AALFPA054267 Aalb-POX18
AALFPA066619 Aalb-POX10
AALFPA073439 Aalb-POX3
AALFPA058573 Aalb-POX12
AALF002755 Aalb-POX16
AALFPA079410 Aalb-POX2
0.20

B

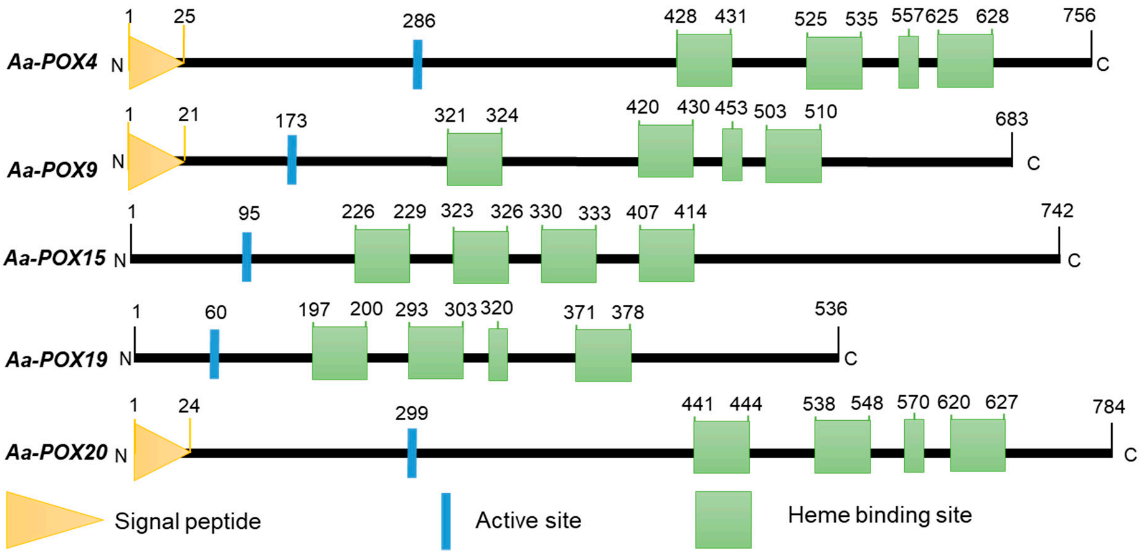
Aa-POX4
1
25
286
428
431
525
535
557
625
628
756
N
C
Aa-POX9
1
21
173
321
324
420
430
453
503
510
683
Aa-POX15
1
95
226
229
323
326
330
333
407
414
742
Aa-POX19
1
60
197
200
293
303
320
371
378
536
Aa-POX20
1
24
299
441
444
538
548
570
620
627
784
Signal peptide
Active site
Heme binding site

Figure S3

A

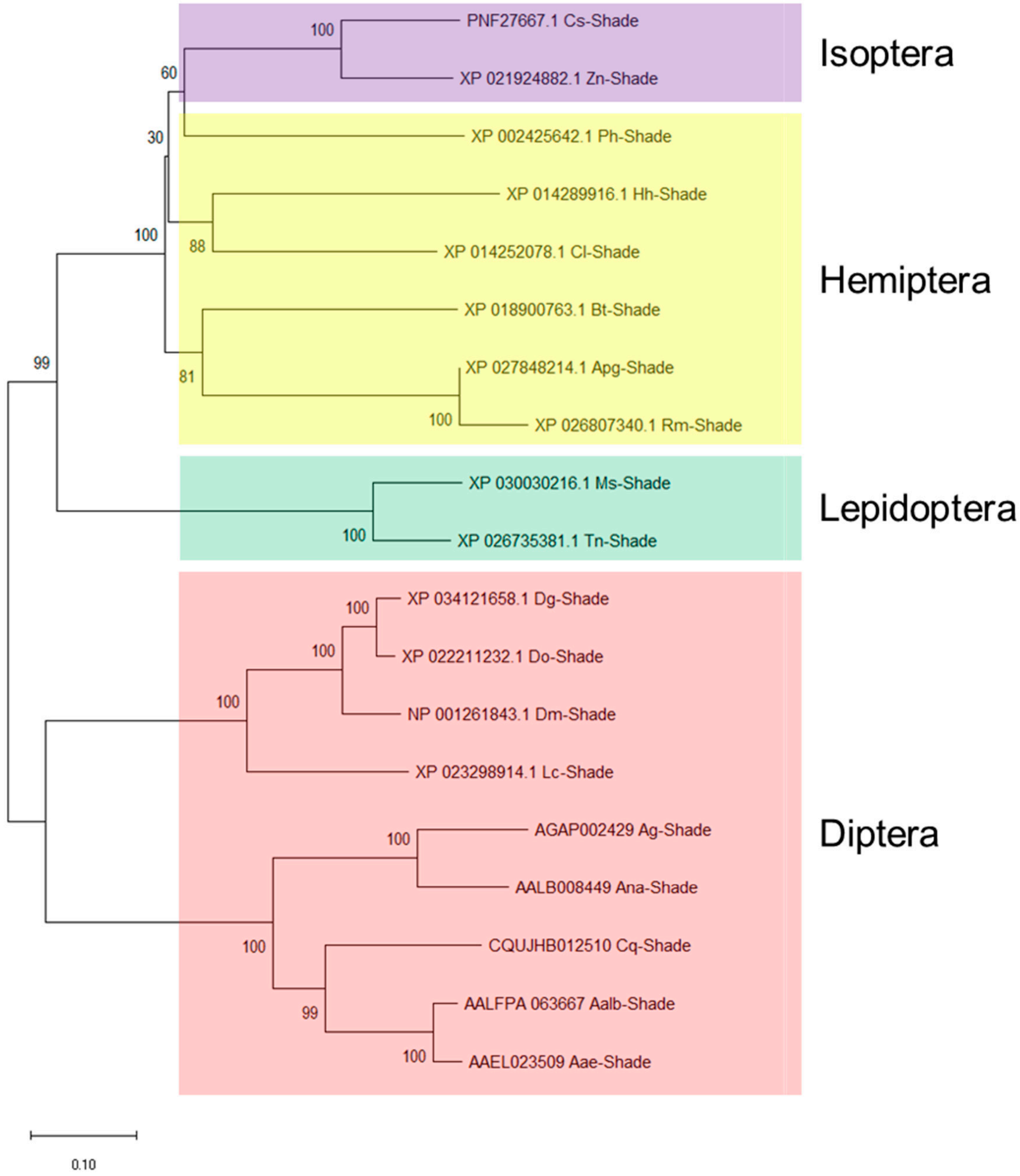

PNF27667.1 Cs-Shade
XP 021924882.1 Zn-Shade
XP 002425642.1 Ph-Shade
XP 014289916.1 Hh-Shade
XP 014252078.1 Cl-Shade
XP 018900763.1 Bt-Shade
XP 027848214.1 Apg-Shade
XP 026807340.1 Rm-Shade
XP 030030216.1 Ms-Shade
XP 026735381.1 Tn-Shade
XP 034121658.1 Dg-Shade
XP 022211232.1 Do-Shade
NP 001261843.1 Dm-Shade
XP 023298914.1 Lc-Shade
AGAP002429 Ag-Shade
AALB008449 Ana-Shade
CQUJHB012510 Cq-Shade
AALFPA 063667 Aalb-Shade
AAEL023509 Aae-Shade
Isoptera
Hemiptera
Lepidoptera
Diptera
0.10

B

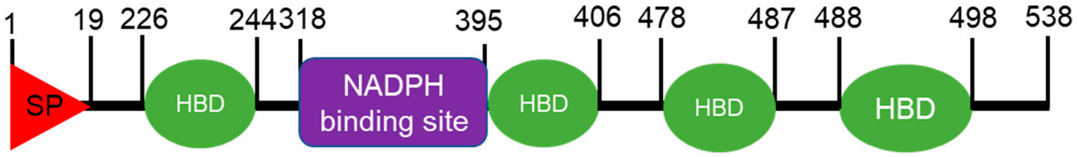

1
19
226
244318
395
406
478
487
488
498
538
SP
HBD
NADPH binding site
HBD
HBD
HBD

Figure S4

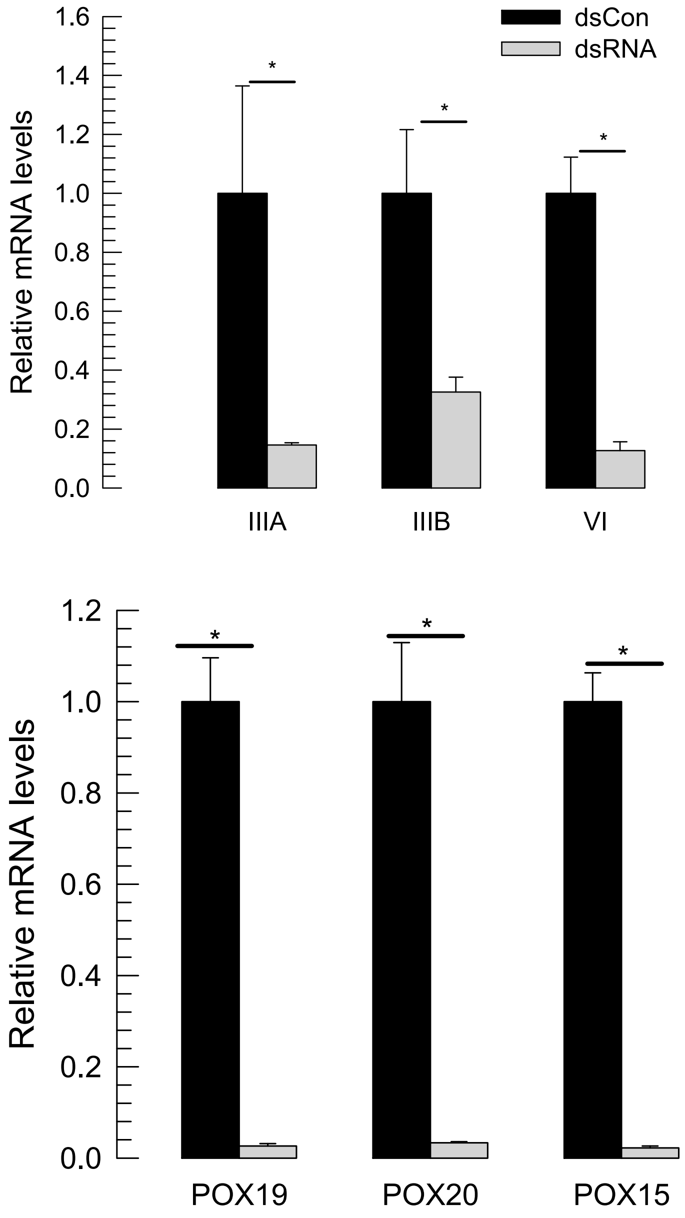
dsCon
dsRNA
Relative mRNA levels
1.6
1.4
1.2
1.0
0.8
0.6
0.4
0.2
0.0
IIIA
IIIB
VI
Relative mRNA levels
1.2
1.0
0.8
0.6
0.4
0.2
0.0
POX19
POX20
POX15

Figure S5

A

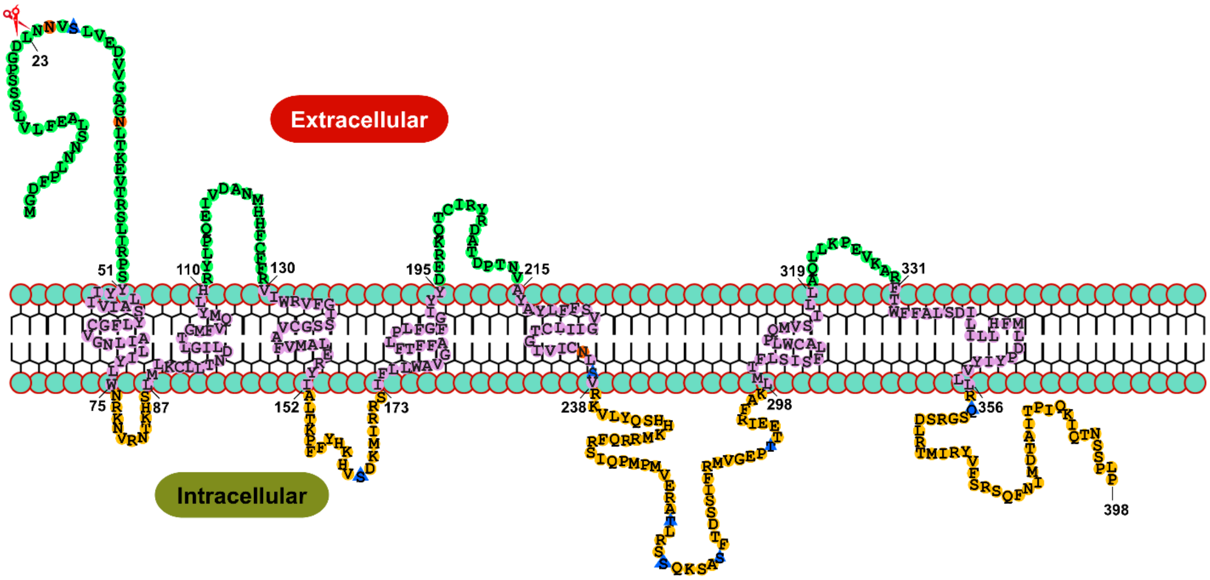

Extracellular
Intracellular
23
51
75
87
110
130
152
173
195
215
238
298
319
331
356
398

B

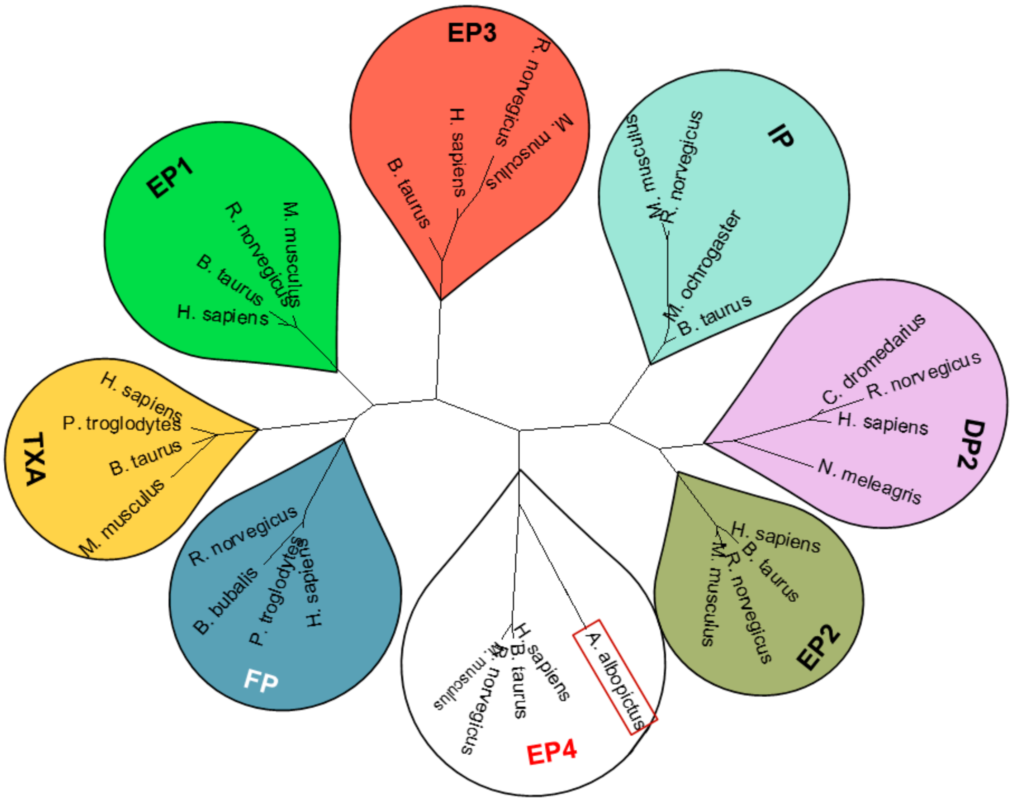

EP3
R. norvegicus
M. musculus
H. sapiens
B. taurus
EP1
M. musculus
R. norvegicus
B. taurus
H. sapiens
IP
M. musculus
R. norvegicus
M. ochrogaster
B. taurus
TXA
H. sapiens
P. troglodytes
B. taurus
M. musculus
DP2
C. dromedarius
R. norvegicus
H. sapiens
N. meleagris
FP
R. norvegicus
B. bubalis
P. troglodytes
H. sapiens
EP4
H. sapiens
B. taurus
R. norvegicus
M. musculus
A. albopictus
EP2
H. sapiens
B. taurus
R. norvegicus
M. musculus

Figure S6

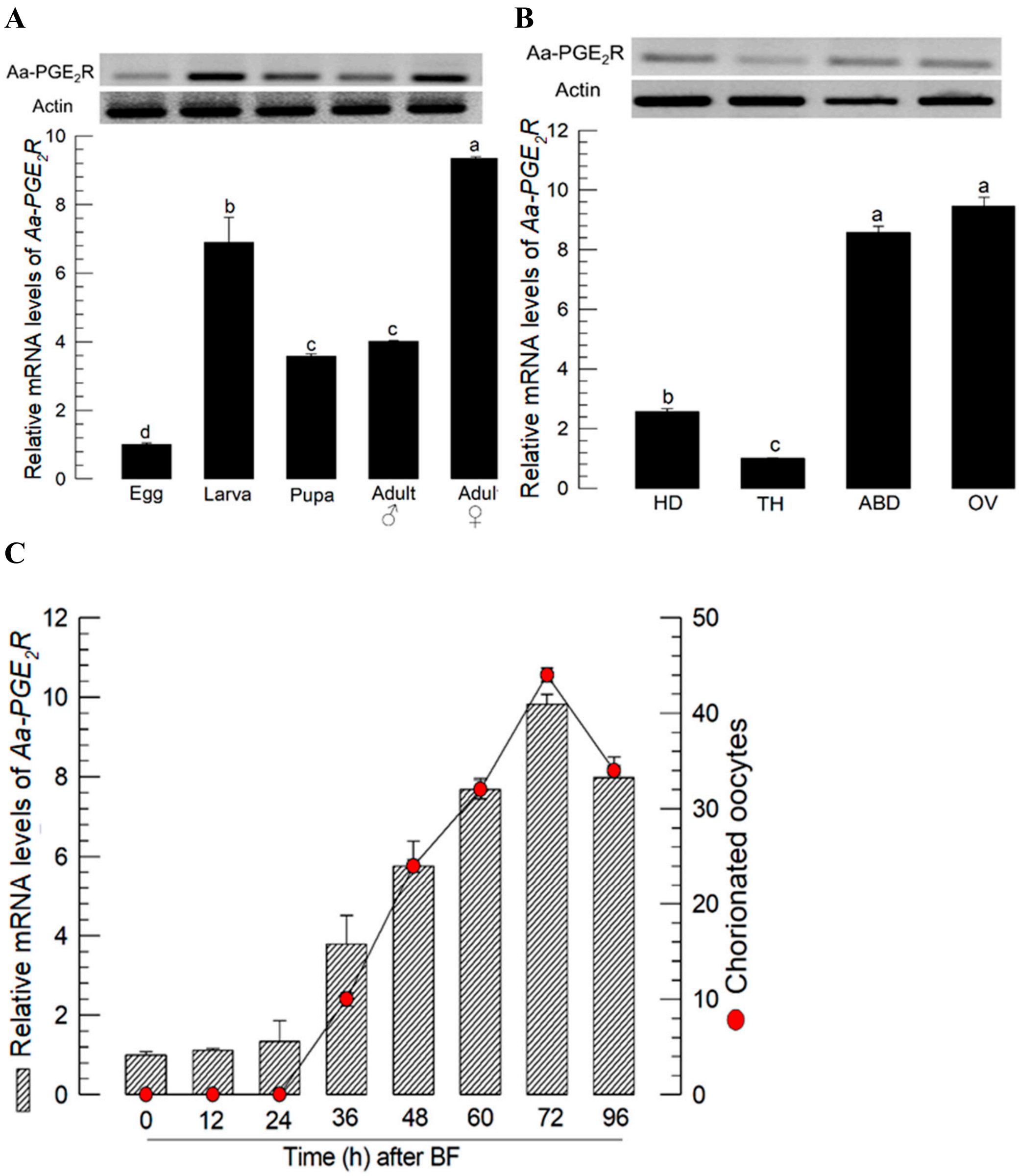
A
Aa-PGE2R
Actin
Relative mRNA levels of Aa-PGE2R
Egg
Larva
Pupa
Adult ♂
Adult ♀
B
Aa-PGE2R
Actin
HD
TH
ABD
OV
C
Chorionated oocytes
Time (h) after BF

Figure S7

A

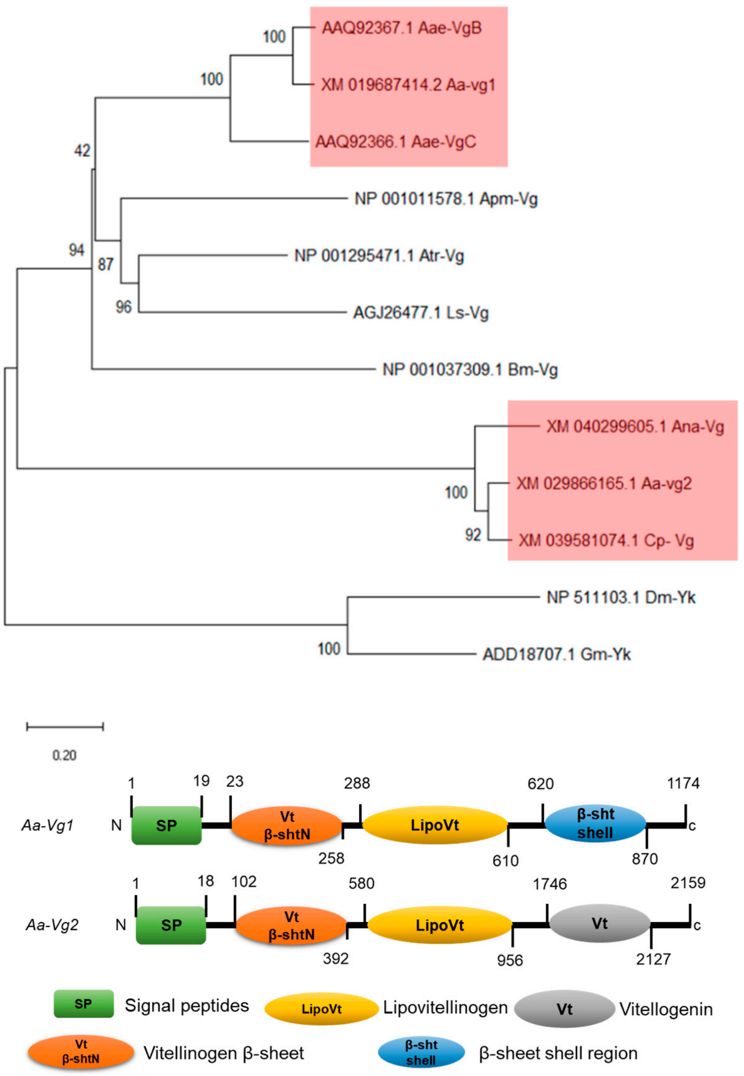

100
AAQ92367.1 Aae-VgB
100
XM 019687414.2 Aa-vg1
AAQ92366.1 Aae-VgC
42
NP 001011578.1 Apm-Vg
94
87
NP 001295471.1 Atr-Vg
96
AGJ26477.1 Ls-Vg
NP 001037309.1 Bm-Vg
XM 040299605.1 Ana-Vg
100
XM 029866165.1 Aa-vg2
92
XM 039581074.1 Cp- Vg
NP 511103.1 Dm-Yk
100
ADD18707.1 Gm-Yk
0.20
Aa-Vg1
N
SP
Vt β-shtN
LipoVt
β-sht shell
c
1 19 23 288 620 1174
258 610 870
Aa-Vg2
N
SP
Vt β-shtN
LipoVt
Vt
c
1 18 102 580 1746 2159
392 956 2127
Signal peptides
Lipovitellinogen
Vitellogenin
Vitellinogen β-sheet
β-sheet shell region

B

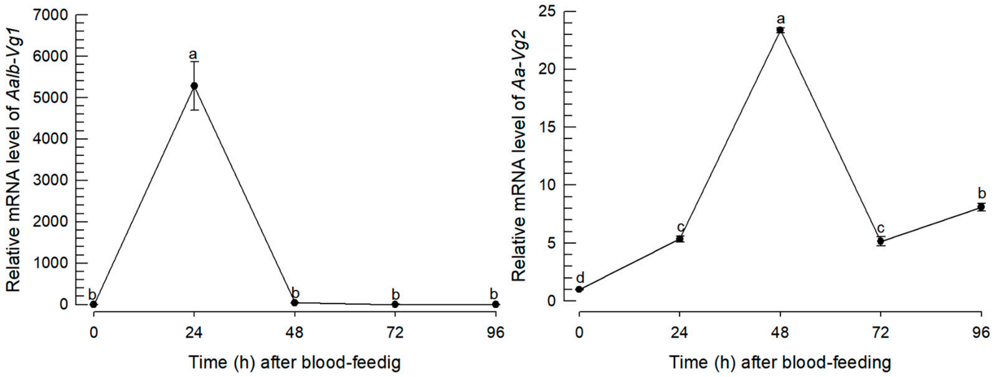

Relative mRNA level of Aalb-Vg1
7000
6000
5000
4000
3000
2000
1000
0
a
b
b
b
b
0
24
48
72
96
Time (h) after blood-feedig
Relative mRNA level of Aa-Vg2
25
20
15
10
5
0
a
d
c
c
b
Time (h) after blood-feeding

Figure S8
